# Overcoming the Barriers to Greater Public Engagement

**DOI:** 10.1371/journal.pbio.1001761

**Published:** 2014-01-14

**Authors:** Ian M. Devonshire, Gareth J. Hathway

**Affiliations:** Laboratory of Developmental Nociception, School of Life Sciences, Nottingham University Medical School, Queen's Medical Centre, Nottingham, United Kingdom; King's College London, United Kingdom

## Abstract

Integrating science communication training into an undergraduate research project encourages greater academic involvement in public engagement, maximizes audience size, and provides high-quality research data.

*This Community Page is part of the Public Engagement in Science series*

The concept of public engagement (PE) in science has evolved steadily over the last thirty years. Early PE activities were often delivered didactically in a one-way flow of information, largely built on the belief that if only the public were told about science, then deficiencies in their understanding could be corrected, and support for scientific funding would improve [1]. Over time, PE has become more interactive, promoting a mutually beneficial goal of creating a more scientifically and technologically literate society by portraying science as an open and transparent field capable of responding to the needs of society [2]–[5]. Indeed, thanks to an explosion in the number and variety of individual PE events—each shaped by the particular branch of science, type of institution, purpose of the activity, and intended audience [2],[6]—it is now easier to categorise PE based on the stated purpose rather than the specific activity [2]. Typically, most activities are conducted by only a small number of scientists, many of whom consider PE a moral and scientific imperative [7],[8]. Recent estimates indicate that 50% of PE activities at higher education institutions are carried out by only 5% of scientists [9]. However, this skewed distribution appears destined to change. All major science funding agencies in the US and Europe now require applicants to describe the PE activities that they will undertake to publicise their research in the wider community [10]. Higher education institutions too are becoming aware of PE's broad mutual benefits for scientists and society [11]–[16]. Accordingly, universities, especially in the UK, have begun signing up to an “engagement manifesto” to declare a commitment to sharing knowledge, resources, and skills with all sections of society [17].

The engagement manifesto and other such guides and initiatives [6],[13],[17],[18] have inevitably raised awareness of what PE can achieve, but it's not always clear whether efforts made to organise PE activities are truly valued by an institution when placed alongside the priorities of teaching and research. Given the demands placed on academics, including the near constant pursuit of funding, it is not surprising that lack of time is one of the major barriers to greater involvement in PE [9],[19]–[21]. Despite calls for better co-ordination between funding agencies and universities to provide a structure for more effective PE [19], there has been little effort to address this problem. Practical guidance must be given alongside PE initiatives—and incentives offered—in order to support and encourage researchers to participate in the kind of engagement increasingly expected of them [19].

## A New Model of Engagement

Our research group has devised a new model of PE for higher education institutions, which we refer to as BrainLab, that aims to help researchers overcome barriers to participating in PE whilst also addressing the call by the American Association for the Advancement of Science to improve undergraduate and postgraduate students' ability to effectively communicate science to diverse audiences [12]. The central features of BrainLab are (1) the integration of a science communication course into the undergraduate syllabus, (2) the delivery of science workshops by academics and undergraduate students (henceforth “students”) in local schools, and (3) the collection of data for use by academics in basic research. The BrainLab model benefits all parties involved: local education authorities receive a greater number of school visits from passionate, enthusiastic scientist visitors, students gain skills and experience that improve their future employment prospects, and academics gain both teaching credit and research data that can contribute towards publications.

One approach to reducing the extra time needed to plan and deliver PE activities [19],[22] is to incorporate a science communication course into the formal undergraduate (or post-graduate) syllabus, so academics can receive teaching credit for engagement activities with no additional time being expended over and above normal teaching loads. Several such science communication training programmes have recently been successfully piloted on a variety of scales, budgets, and time frames [14],[23],. The teaching load may also be shared with non-academic staff that might be involved in existing university–community partnerships. Indeed, many UK universities now have a team of dedicated staff for widening participation in higher education—a widening participation team (WPT) [25],[26]—and these dedicated staff commonly provide a range of science communication training for both staff and students.

Another way to encourage researchers to participate in PE is by incorporating an experimental component into classroom-based science workshops that allows collection of high-quality data that could in turn contribute to diverse academic publications [27]. Research conducted under such circumstances could, for instance, examine the effect of different teaching styles in a given discipline and, therefore, be of particular interest to academics with an interest in pedagogy. Such studies can allow researchers to address a range of topical issues in fledgling fields such as neuroeducation [28],[29], as well as in science education more generally [30],[31]. It is also important to collect feedback after PE on whether objectives were achieved: such feedback can be collected with short, easy-to-complete quantitative and/or qualitative evaluations from students, pupils, and teachers [10]. This information can be used to justify ongoing/wider PE involvement or to improve the activity. The BrainLab model of PE also encourages collaboration or data sharing with central higher education institutions and/or social science departments [32],[33], which could facilitate studies of how PE influences issues as diverse as how school children regard science and why fewer pupils from disadvantaged backgrounds but with equal academic ability apply for university places [34]. Of course, no matter how worthy the goals of research conducted in conjunction with PE, researchers must take care not to exploit participants by, for example, making participation contingent upon lengthy and disruptive data collection. And all research, no matter how trivial, should also be cleared by appropriate ethical review boards.

## Combining Training, Delivery, and Research

BrainLab combines science communication training with a research project for final (third) year neuroscience students at a large UK university. The eight-month programme serves as the “honours year” project for up to ten students and constitutes 40% of their final year grade; the end result is a 10,000-word dissertation including a literature review and data analysis component. Each student is trained in the design and delivery of age-appropriate material and required to prepare a 90-minute science workshop on a neuroscientific theme of their choosing suitable for school pupils aged 9–10 years. Themes chosen in the past have included memory, neurotransmission, and brain diseases. Each student is responsible for leading their own workshop, with fellow students providing classroom support. The academic conducts an introductory session with the pupils that takes place one week before the student's 90-minute workshop. This session lasts 30–60 minutes and is designed to gauge current understanding, convey some basic information about the brain using, for example, games or medical case stories, and enthuse pupils ahead of the following week's workshop. The format of this introductory session is highly flexible and, thus, can be adapted to suit the experience level of the academic involved.

Training is divided between the WPT at the university and the academic supervisors. Although elements of the training are formalised, including one-to-one meetings, we find that discussions held as an entire group, facilitated by the lead academic, best aid the development of students' workshop ideas and content. Thus, each student retains overall responsibility for his/her own workshop, the theme chosen, planning, delivery, and writing the dissertation and yet, at early stages of the project, benefits from support and creative input from the group as a whole. Collaborating with non-academic university staff members offers access to a pre-established network of local schools, saving academics the time-consuming task of finding appropriate and willing audiences (in addition to the WPT, we have also been aided in the recruitment of schools by IntoUniversity, a UK charity that provides university experience and academic support for school pupils in disadvantaged areas).

A description of our most recent BrainLab presentation best illustrates how our approach can be implemented by other institutions. The research topic investigated was the effect of learning games that involve risk-taking on the subsequent retention of information. Though the scientific and education literature includes many reports on this topic [35],[36], it has never been investigated, to the best of our knowledge, in a controlled manner. To address this issue, each student delivered their workshop three times to three separate classes, each time with identical scientific content. In one workshop we included a risk-based learning game in which small groups of school pupils had to risk a number of tokens on answers to multiple-choice science questions posed at specific points throughout the workshop. Groups placing tokens on the correct answer received double the number of tokens back; to provide incentive, it was announced that the team with the most tokens at the end of the game would win a prize (kindly donated by the Dana Foundation). School pupils in the second workshop answered multiple-choice science questions without risking tokens, whilst the third workshop did not involve any questioning. Students took a short pen-and-paper science quiz at the end of all workshops and again one week later in order to measure how much information from the workshops was retained by the pupils. Pupils and their teachers also completed a general evaluation questionnaire after each workshop. Thus, each student collected data from three conditions: risk, no risk, and the control group, which could be pooled between students to assess how the intervention influenced retention of information. This resulted in statistically favourable sample sizes of up to 150 school pupils for each condition. Though a full description of the results is not the primary aim of this article (and is being submitted for publication separately), briefly, we found significantly increased quiz scores one week after the workshop for pupils who had taken part in the risk-based learning games (difference in “risk group” quiz scores one week apart was greater than for both “non risk” and “control” groups; Kruskal-Wallis with Dunn's post-hoc test, overall *n* = 291, *p*<0.01). The results suggest that valuable data can be collected in the course of a PE programme, increasing its impact (see Box 1).

**Box 1. Impact**

BrainLab is aligned with the University of Nottingham's broader initiative to widen participation in higher education by raising the awareness of university life, education, and research in disadvantaged communities [25]. The purpose of collecting quantitative and qualitative feedback from school pupils and teachers was to evaluate the success of training students to deliver professional, engaging, and informative workshops. We also collected students' (anonymous) views of how they thought the course would affect their future careers. Informal discussions at the beginning of the course highlighted a concern about adapting material for school pupils aged 9–10 years old and whether pupils would learn new scientific material in addition to just having fun. High pupil test scores—average end-of-workshop test score 74.01% (standard deviation = 18.79, *n* = 291) ; average score one week later 75.34% (standard deviation = 21.72, *n* = 291) —demonstrate strong learning and high retention one week later, whilst evaluations from teachers (*n* = 17) testify to the success of the training: 100% of teachers surveyed agreed that students delivered material at an age-appropriate level and showed enthusiasm for the material; 94% considered workshops to be well organised; 100% believed their pupils enjoyed the workshops and that they learned new information; and, finally, 100% of teachers would recommend our workshops and delivery method to other schools. A sample of comments made by the teachers serve as a testament to the effort and commitment of the undergraduate students: “The workshops were well-planned, brilliantly delivered, engaging and interactive without losing the academic content. Outstanding! I would definitely recommend” and “A fantastic experience for the children which, without you guys, they would never have had. [We] would love to participate in more please!” Pupil feedback supported the teachers' impressions: between 93% and 96% of pupils (*n* = 328) found the workshops to be fun and interesting, indicated that they learned new information, and wanted us to return again in the future. We also collected anonymous feedback from the students in order to learn how the programme could be improved or changed in the future. 100% of students (*n* = 6) strongly agreed that it was useful academically and useful in terms of future skills and experience, and that they would strongly recommend it to other undergraduates, felt supported throughout, and felt appropriately prepared before hosting workshops.

## Becoming “Pro-Engagement”

One final barrier to greater involvement in PE by academics is cynicism from peers, based on the perception that scientists become involved in PE because their academic performance is under par or else publish less than their colleagues because of their external activities [19]. This is often, and unfairly, referred to as the “Sagan Effect” after the physicist Carl Sagan's pioneering efforts in PE. The name is something of a misnomer, however, given that Sagan averaged one peer-reviewed scientific journal article per month during his career [37]. Indeed, a recent study has shown that academics who are involved in PE have a higher bibliographic index than their non-engaging colleagues, an index that increases with greater PE activity [38]. This finding supports the notion that PE can be mutually beneficial: enabling scientists to see their research from new perspectives whilst fulfilling the particular goal of the event, be it raising aspiration in children, busting common science myths among adults, or improving understanding of a subject currently receiving media attention. Nevertheless, despite both an increased awareness of PE and the latest requirements from funding agencies, greater involvement in PE may depend on a change in academic structures: top-down initiatives from individual higher education institutions that offer practical guidance, support, and incentives. Arguably, this is already taking place with the creation of WPTs based in universities and with the recognition that PE activities now gain in the UK's Research Excellence Framework, a system for assessing research quality and allocating research funds in which a range of PE activities can now contribute to the overall impact of research. However, to avoid PE becoming a perfunctory and impassive box-ticking exercise, we still need an academic community that is “pro-engagement”; academics must have a desire to become involved in PE and have specific outcomes in mind. We believe that PE projects such as BrainLab provide an opportunity for academics, even those with little previous experience, to become more involved in PE and, by working closely with students in setting workshop goals and controlling content, become aware of the benefits of PE and gain teaching credit for their time. In addition to overcoming the major barrier of time constraints in academia, the BrainLab model offers an incentive by incorporating a research component into the engagement activities. Importantly, undergraduate student training will help ensure that the next generation of scientists has the skills to explain important scientific principles in a straightforward and effective manner to the general public.

## Abbreviations

PE: public engagement
WPT: widening participation team

## References

[1] Royal Society (1985) The public understanding of science. Available: http://royalsociety.org/policy/publications/1985/public-understanding-science/. Accessed 10 October 2013.

[2] Davies SR (in press) Constituting public engagement: meanings and genealogies of PEST in two U.K. studies. Sci Commun

[3] Parliamentary Office of Science and Technology (2003) Science and society: three years on. Available: http://www.parliament.uk/documents/post/13-may-proceedings.pdf. Accessed 10 October 2013.

[4] House of Lords Select Committee on Science and Technology (2000) Science and society—third report. Session 1999–2000. Available: http://www.publications.parliament.uk/pa/ld199900/ldselect/ldsctech/38/3801.htm. Accessed 10 October 2013.

[5] Gregory J, Lock SJ (2008) The evolution of public understanding of science: public engagement as a tool of science policy in the UK. Sociology Compass 2: 1252–1265.

[6] Department for Business Innovation and Skills (2010) Science for all: a report and action plan from the Science for All Expert Group. Available: http://www.bis.gov.uk/wp-content/uploads/2010/02/Science-For-All-Report.pdf. Accessed 10 October 2013.

[7] Marincola E (2003) Research advocacy: why every scientist should participate. PLoS Biol 1: e71 doi:10.1371/journal.pbio.0000071 14691543 10.1371/journal.pbio.0000071 PMC300681

[8] Friedman DP (2008) Public outreach: a scientific imperative. J Neurosci 28: 11743–11745. 19005034 10.1523/JNEUROSCI.0005-08.2008 PMC6671662

[9] Ecklund EH, James SA, Lincoln AE (2012) How academic biologists and physicists view science outreach. PLoS ONE 7: e36240 doi:10.1371/journal.pone.0036240 22590526 10.1371/journal.pone.0036240 PMC3348938

[10] Lok C (2010) Science funding: science for the masses. Nature 465: 416–418. 20505707 10.1038/465416a

[11] National Academy of Sciences (2007) Rising above the gathering storm: energizing and employing America for a brighter economic future. Available: http://www.nap.edu/catalog.php?record_id=11463. Accessed 8 July 2013.

[12] American Association for the Advancement of Science (2011) Vision and change in undergraduate biology education: a view for the 21st century. Available: http://visionandchange.org/files/2011/03/Revised-Vision-and-Change-Final-Report.pdf. Accessed 8 July 2013.

[13] Joseph Rowntree Foundation (2012) How can universities support disadvantaged communities? Available: http://www.jrf.org.uk/sites/files/jrf/disadvantaged-communities-and-universities-full.pdf. Accessed 8 July 2013.

[14] Beck MR, Morgan EA, Strand SS, Woolsey TA (2006) Volunteers bring passion to science Outreach. Science 314: 1246–1247. 17124309 10.1126/science.1131917

[15] Foster KM, Bergin KB, McKenna AF, Millard DL, Perez LC, et al (2010) Science education. Partnerships for STEM education. Science 329: 906–907. 20724621 10.1126/science.1191040

[16] Irwin A (1999) Science and citizenship. In: Scanlon E, Whitelegg E, Yates S, editors. Communicating science: contexts and channels. London: Routledge. pp. 14–36.

[17] National Co-ordinating Centre for Public Engagement (2010) Manifesto for public engagement. Available: http://www.publicengagement.ac.uk/why-does-it-matter/manifesto. Accessed 8th July 2013.

[18] Duncan S, Spicer S (2010) The engaging researcher. Cambridge (UK): Careers Research and Advisory Centre. Available: http://www.vitae.ac.uk/CMS/files/upload/The_engaging_researcher_2010.pdf. Accessed 12 December 2013.

[19] The Royal Society (2006) Survey of factors affecting science communication by scientists and engineers. Available: http://royalsociety.org/Content.aspx?id=5232. Accessed 8 July 2013.

[20] Nature Neuroscience Editorial (2009) Encouraging science outreach. Nat Neurosci 12: 665. 10.1038/nn0609-665 19471258

[21] Abrahamsen L (2004) Learning partnerships between undergraduate biology students and younger learners. Microbiol Educ 5: 21–29. 23653554 10.1128/jmbe.v5.74 PMC3633130

[22] National Science Board (2004) Science and engineering indicators 2004. Available: http://www.nsf.gov/statistics/seind04/toc.htm. Accessed 8 July 2013.

[23] Foy JG, Feldman M, Lin E, Mahoney M, Sjoblom C (2006) Neuroscience workshops for fifth-grade school children by undergraduate students: a university-school partnership. CBE Life Sci Educ 5: 128–136. 17012203 10.1187/cbe.05-08-0107 PMC1634799

[24] Romero-Calderon R, O'Hare ED, Suthana NA, Zeeland AASV, Rizk-Jackson A, et al (2012) Project Brainstorm: using neuroscience to connect college students with local schools. PLoS Biol 10: e1001310 doi:10.1371/journal.pbio.1001310 22529746 10.1371/journal.pbio.1001310 PMC3328426

[25] Higher Education Funding Council for England, Office for Fair Access (2013) National strategy for access and student success: interim report to the Department for Business, Innovation and Skills. Available: http://www.hefce.ac.uk/media/hefce/content/news/news/2013/NatStrat_interim_report.pdf. Accessed 8 July 2013.

[26] House of Commons Committee on Public Accounts (2009) Widening participation in higher education. Fourth report of session 2008–09. Available: http://www.publications.parliament.uk/pa/cm200809/cmselect/cmpubacc/226/9780215526557.pdf. Accessed 10 October 2013.

[27] Devonshire IM, Davis J, Fairweather S, Highfield L, Thaker C, et al.. (2013) Maximising the benefits of outreach: training undergraduates in science communication and raising aspirations whilst performing research [abstract]. British Neuroscience Association Biennial Meeting; 7–10 Apr 2013; London, UK.

[28] Devonshire IM, Dommett EJ (2010) Neuroscience: viable applications in education? Neuroscientist 16: 349–356. 20817916 10.1177/1073858410370900

[29] Della Salla S, Anderson M, editors (2012) Neuroscience in education: the good, the bad and the ugly. Oxford: Oxford University Press.

[30] Hines PJ, Mervis J, McCartney M, Wible B (2013) Grand challenges in science education. Plenty of challenges for all. Science 340: 290–291. 23599475 10.1126/science.340.6130.290

[31] Klahr D, Zimmerman C, Jirout J (2011) Educational interventions to advance children's scientific thinking. Science 333: 971–975. 21852489 10.1126/science.1204528

[32] Laursen S, Liston C, Thiry H, Graf J (2007) What good is a scientist in the classroom? Participant outcomes and program design features for a short-duration science outreach intervention in K-12 classrooms. CBE Life Sci Educ 6: 49–64. 17339394 10.1187/cbe.06-05-0165 PMC1810206

[33] Ashby J, Wood C (2010) Lessons in learning: primary schools, universities and museums. A best-practice guide by University College London. London: UCL Museums and Collections.

[34] UK Department for Education and Skills (2003) Widening participation in higher education. Available: http://www.bis.gov.uk/assets/BISCore/corporate/MigratedD/publications/E/EWParticipation.pdf. Accessed 8 July 2013.

[35] Howard-Jones P (2011) Toward a science of learning games. Mind Brain Educ 5: 33–41.

[36] Evans D (2013 February 22) Can gambling be useful? You bet. Times Educ Suppl

[37] Shermer MB (2002) This view of science: Stephen Jay Gould as historian of science and scientific historian, popular scientist and scientific popularizer. Soc Stud Sci 32: 489–524. 12503565 10.1177/0306312702032004001

[38] Jensen P, Rouquier JB, Kreimer P, Croissant Y (2008) Scientists who engage with society perform better academically. Sci Public Pol 35: 527–541.

